# Low-Cost Technology-Aided Programs for Supporting People With Motor, Visual, and Intellectual Disabilities in Functional Forms of Occupation and Communication: Proof-of-Concept Study

**DOI:** 10.2196/44239

**Published:** 2023-03-24

**Authors:** Giulio E Lancioni, Nirbhay N Singh, Mark F O’Reilly, Jeff Sigafoos, Gloria Alberti, Valeria Chiariello, Lorenzo Desideri, Serafino Buono

**Affiliations:** 1 Department of Neuroscience and Sense Organs University of Bari Bari Italy; 2 Department of Psychiatry and Health Behavior Augusta University Augusta, GA United States; 3 College of Education University of Texas at Austin Austin, TX United States; 4 School of Education Victoria University of Wellington Wellington New Zealand; 5 Lega F. D’Oro Research Center Osimo Italy; 6 AIAS Bologna Onlus Bologna Italy; 7 Oasi Research Institute - IRCCS Troina Italy

**Keywords:** technology, smartphone, tablet, motor impairment, visual impairment, intellectual disability, leisure, communication, stories

## Abstract

**Background:**

People with motor, visual, and intellectual disabilities may have serious problems in independently accessing various forms of functional daily occupation and communication.

**Objective:**

The study was aimed at developing and assessing new, low-cost technology-aided programs to help people with motor or visual-motor and intellectual disabilities independently engage in functional forms of occupation and communication with distant partners.

**Methods:**

Two programs were set up using a smartphone interfaced with a 2-switch device and a tablet interfaced with 2 pressure sensors, respectively. Single-subject research designs were used to assess (1) the first program with 2 participants who were blind, had moderate hand control, and were interested in communicating with distant partners through voice messages; and (2) the second program with 2 participants who possessed functional vision, had no or poor hand control, and were interested in communicating with their partners through video calls. Both programs also supported 2 forms of occupational engagement, that is, choosing and accessing preferred leisure events consisting of songs and music videos, and listening to brief stories about relevant daily topics and answering questions related to those stories.

**Results:**

During the baseline phase (when only a conventional smartphone or tablet was available), 2 participants managed sporadic access to leisure or leisure and communication events. The other 2 participants did not show any independent leisure or communication engagement. During the intervention (when the technology-aided programs were used), all participants managed to independently engage in multiple leisure and communication events throughout the sessions and to listen to stories and answer story-related questions.

**Conclusions:**

The findings, which need to be interpreted with caution given the nature of the study and the small number of participants, seem to suggest that the new programs may be viable tools for helping people with motor or visual-motor and intellectual disabilities independently access leisure, communication, and other forms of functional engagement.

## Introduction

### Background

People with extensive motor disabilities or combinations of motor disabilities and blindness may have serious problems in independently accessing various forms of functional daily occupation [1-5]. The problems may be even more severe when people present with intellectual disability in addition to motor and visual impairments [6-12]. In the latter case, people may not be able to control leisure events such as music, videos, and comedy because of difficulties in reaching and managing common tools used for playing those events (eg, music devices, computers, and television) [6,10,13]. They may find it arduous or impossible to communicate with relevant partners not present in the immediate context because of difficulties in handling a telephone call or sending an SMS text or voice message or reading an incoming message [14-16]. Similarly, they may not succeed in engaging in common daily activities (eg, cooking and cleaning) [8,17,18] and may also find it challenging to participate in simple cultural and cognitive activities (eg, listening to brief stories and answering questions related to them) [19-22].

The possibility of setting up effective programs to help people with disabilities improve their situation and gain some level of independence is increasingly viewed as closely connected to the use of assistive technology solutions [3,23-27]. For example, a variety of such solutions have been developed to support programs aimed at helping (1) people with blindness manage Braille reading and orientation and mobility [23,24] and (2) people with pervasive motor disabilities manage leisure and communication via eye gazing [26,27]. Assistive technology solutions have also been developed to support people who present with sensory, motor, and intellectual disabilities (people who could hardly benefit from the technology solutions developed for individuals with blindness or individuals with pervasive motor impairment) [10,11,28-34].

Some of these last technology solutions were aimed at promoting leisure and communication with distant partners [35,36] or leisure, communication with distant partners, and functional activities [37]. For example, Lancioni et al [35] worked with 6 participants who presented with serious motor and sensory impairments and moderate intellectual disability. The technology used with 4 participants consisted of a tablet with the Android operating system, SIM (subscriber identity module) card, proximity sensor, multimedia player, internet connection, Google account, and the WhatsApp Messenger and MacroDroid apps. Every session started with the tablet sequentially illuminating and verbalizing the names of 2 pictures (choice areas) representing leisure and communication (SMS text messaging), respectively. The participants could select either picture (area) by approaching with their hand the proximity sensor of the tablet while that picture (area) was illuminated. Selection of an area led the tablet to present different alternatives within that area, such as different types of music and videos or different communication partners. If the participants chose a leisure alternative, the tablet presented specific options that could be accessed. If the participants chose the communication alternative (a communication partner), the tablet presented various messages that could be sent to that partner. For 2 participants who could not use the aforementioned hand response due to their extensive motor impairment, a smartphone was available on their wheelchair’s headrest. This allowed them to make their choices by turning their head toward the smartphone, thus activating the proximity sensor of the smartphone.

Lancioni et al [37] worked with 5 participants who presented with motor, visual, and intellectual disabilities. The technology included (1) a smartphone with the Android operating system, SIM card, internet connection, Google account, and MacroDroid app, and (2) 8 mini voice-recording devices. Each device contained a recorded verbal message that was uttered as the participant applied a simple hand pressure on the device. The message consisting of a request for a leisure event or a telephone call activated the smartphone’s Google Assistant, which in turn led the smartphone to present a leisure event or start a call. Periods with leisure events and telephone calls were interspersed with daily activity periods. During the latter periods, smartphone’s instructions for the activity steps were available.

The results of the aforementioned studies showed that the technology-aided programs were suited for leading the participants to independently manage leisure and communication and possibly combine them with daily activities. On the basis of these results, one can find new motivation to develop additional, upgraded programs that (1) would target leisure, communication, and other forms of useful engagement; (2) would be practical and easily accessible in terms of technology components and cost; and (3) would suit participants with limited motor abilities.

### Objectives

This study was an effort to develop 2 new, low-cost technology-aided programs, namely, programs relying on technology components that are commercially available (off-the-shelf), easy to operate and maintain, and have costs of less than US $1000 [38,39]. The first program involved a smartphone linked via Bluetooth to a 2-switch device and was assessed with 2 participants who were blind, had moderate hand control, and were interested in communicating with distant partners through voice messages. The second program involved a tablet linked via a Bluetooth interface to 2 pressure sensors and was assessed with 2 participants who possessed functional vision, had no or poor hand control, and were interested in communicating with their partners through video calls. In addition to leisure and communication, both programs sought to support a third (functional) type of occupation that would (1) be feasible for the participants’ motor, sensory, and intellectual conditions and (2) replace the conventional daily activities (not suitable for these participants), which had been used in previous programs [37]. This third occupation consisted of listening to brief stories dealing with relevant daily topics (eg, sport, geography, music, and food) and answering questions related to those stories.

## Methods

### Participants

The participants are here identified through the pseudonyms of Aubrey, Joseph, Collins, and Dylan. Aubrey and Joseph were the participants who used the first program while Collins and Dylan were the participants who used the second program. Table 1 summarizes their condition by reporting their chronological age, their visual and motor impairments, and their age equivalents for receptive and expressive communication as measured via the second edition of the Vineland Adaptive Behavior Scales [40,41]. Their chronological age varied between 25 (Dylan) and 53 (Aubrey) years. Their Vineland age equivalents on receptive and expressive communication were between 5 years and 10 months and 7 years and 1 month, and between 4 years and 5 months and 6 years and 5 months, respectively. Their communication occurred verbally. Their utterances, however, were not clear and easy to understand for people not familiar with them. They were attending rehabilitation and care centers. The psychological records of those centers indicated that the intellectual disability levels of Joseph, Collins, and Dylan were rated to be in the moderate range, whereas that of Aubrey was reported to be in the mild to moderate range.

**Table 1:** Participants’ pseudonyms, chronological age, visual and motor impairments, and Vineland age equivalents for receptive communication and expressive communication.

Participants (pseudonyms)	Chronological age (years)	Visual and motor impairments	Vineland age equivalents^a,b^	
			Receptive communication	Expressive communication
Aubrey	53	Blindness and spastic tetraparesis, with inability to ambulate	7; 1	6; 5
Joseph	46	Blindness and right arm and leg paresis, with ability to ambulate	6; 6	5; 9
Collins	31	Spastic tetraparesis, with lack of hand control and inability to ambulate	5; 10	4; 5
Dylan	25	Spastic tetraparesis, with reduced hand control and need of some support to ambulate	5; 10	4; 7

^a^The age equivalents are based on the Italian standardization of the Vineland scales [40].

^b^The Vineland age equivalents are reported in years (number before the semicolon) and months (number after the semicolon).

The participants were included in the study following a number of criteria. First, they enjoyed having access to leisure events, such as preferred songs, and exchanging voice messages or making video calls with preferred communication partners (eg, family and staff members) not present in their immediate context. Notwithstanding their interest, they were relying on the assistance of staff or caregivers for accessing both leisure and communication events. Second, they had expressed interest in listening to simple stories concerning topics such as sport, daily events, singers, and geography and to answer questions related to those stories. Third, they had also shown eagerness to use the technology systems set up for this study to support their independent access to leisure, communication, and stories and related questions. Their eagerness followed a preliminary familiarization with the systems. Fourth, staff (1) considered technology-aided programs critical to help the participants reach independence in basic areas of daily life, and (2) agreed with the areas targeted within the study, that is, leisure, communication with distant partners, and listening to simple stories and answering questions related to them, as well as with the systems arranged for the participants. Staff had been able to see the systems ahead of the study.

### Ethical Approval and Informed Consent

All participants had gone through a preliminary familiarization step with the technology system available for them (ie, the smartphone and the Bluetooth switch device or the tablet combined with the Bluetooth interface and pressure sensors) and had shown eagerness to use such a system to manage leisure, communication, and stories. Given their moderate or mild to moderate level of intellectual disability, the aforementioned eagerness was considered to be a clear sign of their willingness (consent) to be involved in the study. Even so, due to the fact that they were unable to read and sign a consent form, their legal representatives were involved in the consent process, that is, in reading and signing the consent form for the participants. The study complied with the 1964 Helsinki declaration and its later amendments and was approved by the Ethics Committee of the Lega F. D’Oro, Osimo, Italy (P020320221).

### Setting, Research Assistants, Sessions, Leisure and Communication, and Stories

The participants’ daily context (ie, areas of the rehabilitation and care centers they attended) served as the study setting. Three research assistants were employed for carrying out the study sessions of the 4 participants and for recording the data (discussed later). They were psychology graduates who had experience in implementing technology-aided intervention programs with people with different levels of disabilities and using data recording procedures.

The study included baseline and intervention sessions, which were carried out on an individual basis, once or twice a day, 3-6 days a week. During baseline sessions, the participants had a smartphone or a tablet (see below) and the research assistants invited them to use the device available to access leisure events (music) and communication (audio messages or video calls) and respond to questions related to stories that the smartphone and tablet read. During the intervention sessions, the participants had the technology system set up to help them access leisure and communication and respond to story-related questions. Each session encompassed 4 leisure and communication periods and 3 stories (see below).

The stories (1) concerned a variety of familiar topics, such as sport, singers and other renowned people, animals, geography, and food recipes; (2) were chosen by the research assistants based on the participants’ general abilities and interests; (3) lasted between 2 and 4 minutes based on the topic represented and participant’s interest on such topic; and (4) were taken from YouTube or copied from websites.

### Technology System I

This technology system was developed for the first program and used by Aubrey and Joseph who were blind, had moderate hand control, and were interested in communicating with their preferred, distant partners through voice messages more than through telephone calls. The technology involved a smartphone with the Android operating system combined with a Bluetooth Blue2 switch (a 16 × 7 × 2-cm device encompassing 2 adjacent pressure-sensitive buttons; AbleNet, Inc). The smartphone was equipped with a SIM card, internet connection, and Google account (Alphabet, Inc), and contained the WhatsApp Messenger (Facebook, Inc) and MacroDroid (Jamie Higgins) apps. The MacroDroid served to regulate the smartphone’s functioning in accordance with the intervention conditions and to assist with data recording (see the “Measures and Data Recording” section). The smartphone was also provided with the telephone numbers of the participants’ communication partners. The 2 buttons of the Bluetooth Blue2 switch were discriminated through a smooth and a hairy cover, respectively.

At the start of a session, the smartphone checked whether there were messages for the participants and eventually read those messages. Thereafter, it verbalized the following sentence: “You can listen to music by pressing the smooth button or can send a message by pressing the hairy button.” If the participant pressed the smooth button, the smartphone verbalized at intervals of 2-4 seconds the names of 4 preferred singers (which could be different during the study). If the participant pressed the same (ie, smooth) button after a singer’s name, the smartphone played a song by that singer. At least four songs (which could vary across sessions) were available for each singer. Songs were played for 1.5 minutes [37].

If the participant pressed the hairy button, the smartphone verbalized at intervals of 2-4 seconds the names of 5 preferred communication partners, which included family and staff members. If the participant pressed the same (ie, hairy) button following one of the names, the smartphone (1) got ready to send a voice message on WhatsApp to that name (partner) and (2) asked the participant to speak (verbalize) the message they wanted to send. Once the message had been spoken the participant had to press the same button to send the message and have confirmation that it was sent out. At the end of a song or message sequence, the smartphone automatically repeated the phrase indicating that it was possible to access music or send a message through the pressure buttons provided the time elapsed from the start of that leisure and communication period had not exceeded 3 minutes.

If the time elapsed was more than 3 minutes, the smartphone invited the participant to listen to a brief story presented by the smartphone and then to answer questions related to the story. The stories concerned a variety of topics and lasted between 2 and 4 minutes (see the “Setting, Research Assistants, Sessions, Leisure and Communication, and Stories” section). At the end of a story, the smartphone presented 5 questions about it. For each question, the smartphone gave the participant 2 possible answers and indicated the pressure button to be activated in relation to each answer (questions and answers were programmed by the research assistants). For example, following a story over a particular football team, 1 of the questions could be “Was that player NAME playing as a goalkeeper or as a center-forward? You can press the smooth button for goalkeeper and the hairy button for center-forward.” If the participant gave the wrong answer (ie, pressed the wrong button), the smartphone did not provide any feedback and paused. When the participant gave the correct answer (ie, pressed the correct button), the smartphone said “OK, Correct” and presented the next question. Once all the questions had been answered, the smartphone repeated the phrase indicating that it was possible to access music or send messages through the pressure buttons. The same process continued for the rest of the session, which included 4 leisure and communication periods interspersed with 3 stories each followed by the related questions. After completing the questions for the third story, the smartphone would read any message that had arrived during the session.

### Technology System II

This technology system was developed for the second program and used by Collins and Dylan who possessed functional vision but had no or poor hand control, and were interested in communicating with their partners through video calls. The system involved a tablet with the Android operating system combined with a Bluetooth Encore Plus interface (Leonardo Ausili) linked to 2 pressure sensors. The sensors (ie, 2 Buddy Buttons with a diameter of 6.3 cm; Leonardo Ausili) were placed at the sides of the wheelchair’s headrest (Collins) or on the desk before the participant, about 25 cm apart (Dylan). The tablet (like the smartphone) was equipped with a SIM card, internet connection, and Google account, and contained the WhatsApp Messenger and MacroDroid apps. The tablet was also provided with the telephone numbers of the communication partners and with their prerecorded answers to telephone calls (see below). This system worked as the first one with 4 exceptions. First, at the start of a session and through any of the leisure and communication periods, the tablet’s verbalization was: “You can listen to music by pressing the red button” or “You can call somebody by pressing the green button.” Second, music videos were used instead of songs. Third, video calls were used instead of voice messages. Fourth, the tablet played a prerecorded message of the communication partners if they did not answer a call.

### Experimental Conditions

#### Design and General Procedures

For each pair of participants (ie, Aubrey and Joseph who used the first program, and Collins and Dylan who used the second program), the intervention was introduced according to a multiple probe across-participants design [40]. That is, the second participant of the pair was presented with a larger number of baseline sessions spread over a longer period as a way to control for the impact of variables such as maturation and history [42,43]. For the participants of the second pair, moreover, the baseline was repeated with a consequent break of the intervention period into 2 phases. In essence, each of these 2 participants experienced an ABAB sequence [43]. The baseline (A) phase(s) served to determine whether the participants could use a smartphone or a tablet to access leisure and communication events and to answer questions related to specific stories. The intervention (B) phase(s) focused on the use of the technology system available to the participants. To ensure procedural fidelity (ie, the research assistants’ appropriate application of the baseline and intervention procedural conditions [44]), a study coordinator who had access to video recordings of the sessions provided the research assistants with regular feedback and possible guidance regarding their performance [37].

#### Baseline

During the baseline sessions, the participants sat in front of a desk where they found the smartphone (Aubrey and Joseph) or the tablet (Collins and Dylan), which were not using MacroDroid and thus functioned in the standard manner. At the start of a session with Aubrey and Joseph, the research assistant explained that they could access preferred songs or send a message to preferred communication partners by saying “Hey Google play singer’s NAME or song’s TITLE” or “Hey Google send a voice message on WhatsApp to partner’s NAME” and then speaking the message. They could also answer the questions about stories that the smartphone would read to them by saying “Ok Google write a note” before giving any answer. Thereafter, the research assistant encouraged the participants to ask for a singer or a specific song. If the participants made an unsuccessful request or failed to make any request for 15-20 seconds, the research assistant provided help (ie, made a request for them to minimize any frustration). The song being played would be stopped after about 1.5 minutes in line with what occurred during the intervention (see the “Technology System I” section). Following the end of the song, the research assistant told the participants that they could send a message to a preferred partner. Again, to reduce participants’ frustration, the research assistant provided help after an unsuccessful effort or failure to make an effort for 15-20 seconds. Help consisted of the research assistant uttering the phrase required to ready the Google Assistant about the WhatsApp message to be sent to a partner so the participants could speak out the message and send it to the partner.

Once the first leisure and communication period (ie, a period of about 3 minutes) was over, the research assistant activated the smartphone for the presentation of a story and of questions related to it. The participants were to listen to the story and then answer the questions. If the participants failed to produce the phrase required for answering the first question (ie, “Ok Google write a note”), the research assistant would (1) produce it for them so that they could provide the answer, (2) block the smartphone’s reading of the following questions (to reduce participants’ frustration), and (3) encourage the participants to ask for a new singer or song and then send a new message (thus starting a new leisure and communication period). During this second leisure and communication period, conditions were as during the first. The session then continued with a new story and questions followed by a new leisure and communication period until 4 such periods and 3 stories had occurred.

The baseline conditions for Collins and Dylan matched those described for Aubrey and Joseph with 1 specific exception. That is, they had the opportunity to start telephone calls to preferred partners (rather than sending voice messages) by saying “Hey Google call partner’s NAME**.**” The decision to include audio calls rather than video calls (which would have been even more pleasing for both participants and indeed were used during the intervention) was due to the fact that the Google Assistant available in a standard smartphone or tablet does not allow one to start video calls.

#### Intervention

During the intervention sessions, the 2 pairs of participants used the 2 technology systems, which worked as described above (see the “Technology System I” and “Technology System II” sections). At the start of the sessions, the smartphone read to the participants of the first pair any message that had arrived and then informed them that they could listen to music or send messages using the smooth and hairy button, respectively. The tablet informed the participants of the second pair that they could activate music and video calls using the red and green buddies on the wheelchair’s headrest (Collins) or on the desk (Dylan). A 3-minute time interval was allocated for this leisure and communication period as well as for any of the following 3 periods scheduled within every session. Any leisure event, message, or call started within the 3-minute interval was to be completed irrespective of whether it would extend the interval. At the end of the single leisure and communication periods, the smartphone or tablet read a story and then presented the 5 related questions that the participants had to answer. Following the last story and prior to the start of the last leisure and communication period, the smartphone read any incoming message(s) to the participants of the first pair. At the end of the sessions, the research assistant gave all participants feedback about their answers to the story-related questions, that is, pointed out how many questions they had answered correctly at first attempt.

The initial 4-6 sessions were used as practice sessions. In the beginning, the research assistant relied on verbal and physical guidance to help the participants use the technology system available to access leisure events, send voice messages or make video calls, and answer the story-related questions. Afterward, any form of research assistant’s help was faded out and eventually the participants were to manage the use of the technology system independently. The regular intervention sessions that followed did not include research assistant’s help unless the participant requested for it. Such request was virtually absent.

### Measures and Data Recording

A total of 5 measures were recorded. The first 3 included leisure events (songs and music videos) activated, voice messages sent or video calls made, and correct answers to the story-related questions produced at first attempt (with the first response given to the questions). All these 3 measures implied independence from any research assistant’s help. The other 2 measures were session duration and voice messages received (read by the smartphone). This last measure was recorded only for the first pair of participants. During the intervention sessions, the smartphone and the tablet automatically recorded all the measures via MacroDroid (ie, made a log of all session events and the related times of occurrence for the research assistants to use). During the baseline sessions, the research assistants recorded the measures. Interrater agreement was checked in all baseline sessions with the involvement of a reliability observer in data recording. The percentage of interrater agreement (computed by dividing the number of baseline sessions in which the research assistant and the reliability observer reported the same number of songs or music videos, messages or calls, and correct answers to the story-related questions as well as duration times differing less than 2 minutes by the total number of baseline sessions, and multiplying by 100%) was 100% for all participants.

### Data Analysis

The frequency of leisure events (ie, songs and music videos) accessed and voice messages sent or video calls made, and the percentage of story-related questions answered correctly at first attempt (with the first response given to the questions; see the “Technology System I” section) were presented in graphic form. The differences between the baseline and intervention data values on the single measures of every participant were analyzed through the percentage of non-overlapping data (PND) method [45]. This method verifies the size of the intervention effect by determining the percentage of intervention data points that are above the highest point of the baseline data.

## Results

### Technology System I

**Figure 1:**
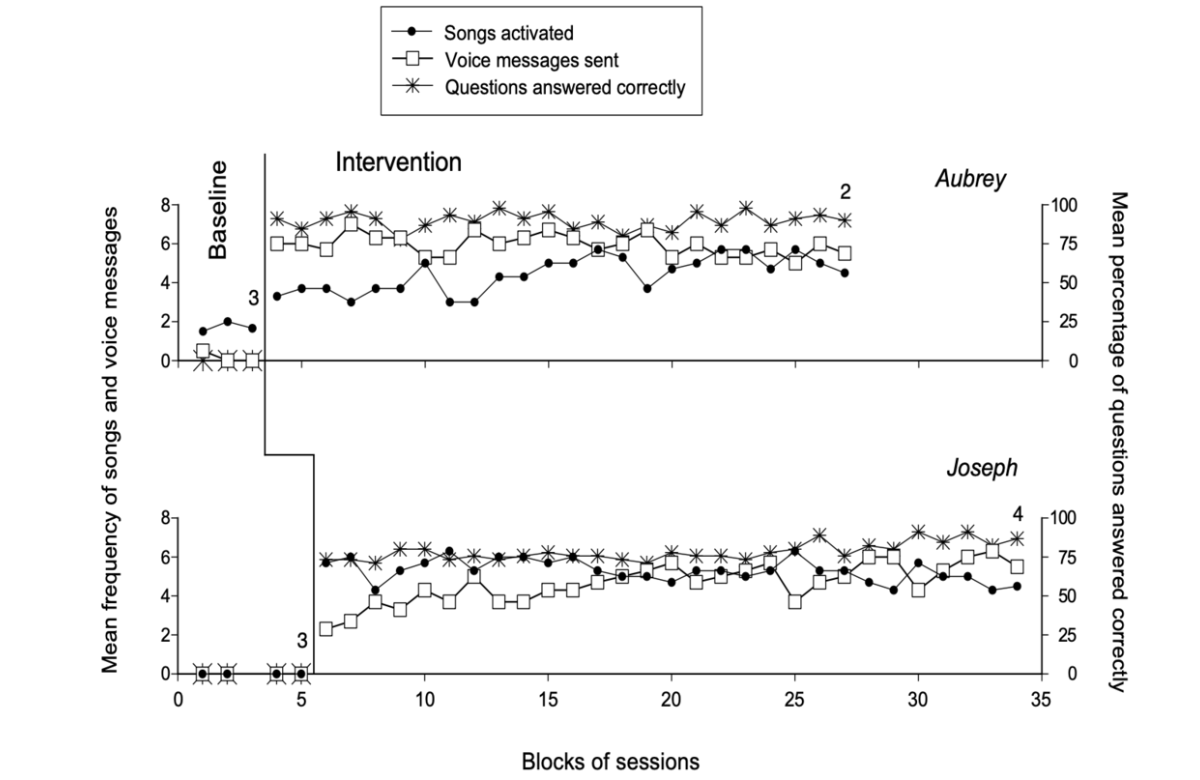
The 2 panels summarize the baseline and intervention data for Aubrey and Joseph. The black circles and empty squares represent the mean frequency of songs activated and voice messages sent per session, respectively, over blocks of 2 sessions during the baseline and blocks of 3 sessions during the intervention. Blocks with different numbers of sessions (ie, at the end of the phases) are marked with a numeral indicating how many sessions are included. The asterisks represent the mean percentage of story-related questions answered correctly at first attempt over the same blocks of sessions.

The 2 panels of Figure 1 summarize the baseline and intervention data for the participants involved in the first program who used Technology System I (ie, Aubrey and Joseph). The black circles and empty squares represent the mean frequency of songs activated and of voice messages sent per session, respectively, over blocks of 2 sessions during the baseline phase and blocks of 3 sessions during the intervention phase. The asterisks represent the mean percentage of story-related questions answered correctly at first attempt over the same blocks of sessions. The practice sessions used at the beginning of the intervention phase are not reported in the figures.

The baseline phase showed that Aubrey (who received 7 sessions over a period of 1 week) activated a mean of 1.7 songs per session, managed to send a total of 1 voice message, and did not answer any story-related question. Joseph (who had 9 sessions spread over a period of more than 2 weeks) failed to access any song, to send any message, and to answer any story-related question. The practice sessions at the beginning of the intervention led the participants to use the technology system, that is, a smartphone in combination with the Bluetooth Blue2 switch, successfully, and to become independent in activating songs, sending voice messages (and accessing incoming messages), and listening to stories and answering the story-related questions. During the 71 (Aubrey) and 88 (Joseph) intervention sessions occurring after the practice sessions, the participants’ mean frequency of songs activated was 4.4 and 5.3 per session, respectively. Their mean frequency of voice messages sent per session was 5.9 and 4.7, respectively. Their mean percentage of correct responses to the story-related questions was 89 and 78, respectively. Their mean frequency of voice messages received was 2.4 and 1.7 per session, respectively. Their mean session duration was about 30 and 26 minutes, respectively.

Comparisons between intervention and baseline data carried out through the PND method on songs activated, voice messages sent out, and correct responses to story-related questions provided indices of 1.0 (ie, all intervention values exceeded the baseline’s highest value) with an exception. The exception concerned the songs activated measure, on which Aubrey had an index of 0.97 (ie, an index that still expresses a strong intervention effect [45]).

### Technology System II

**Figure 2:**
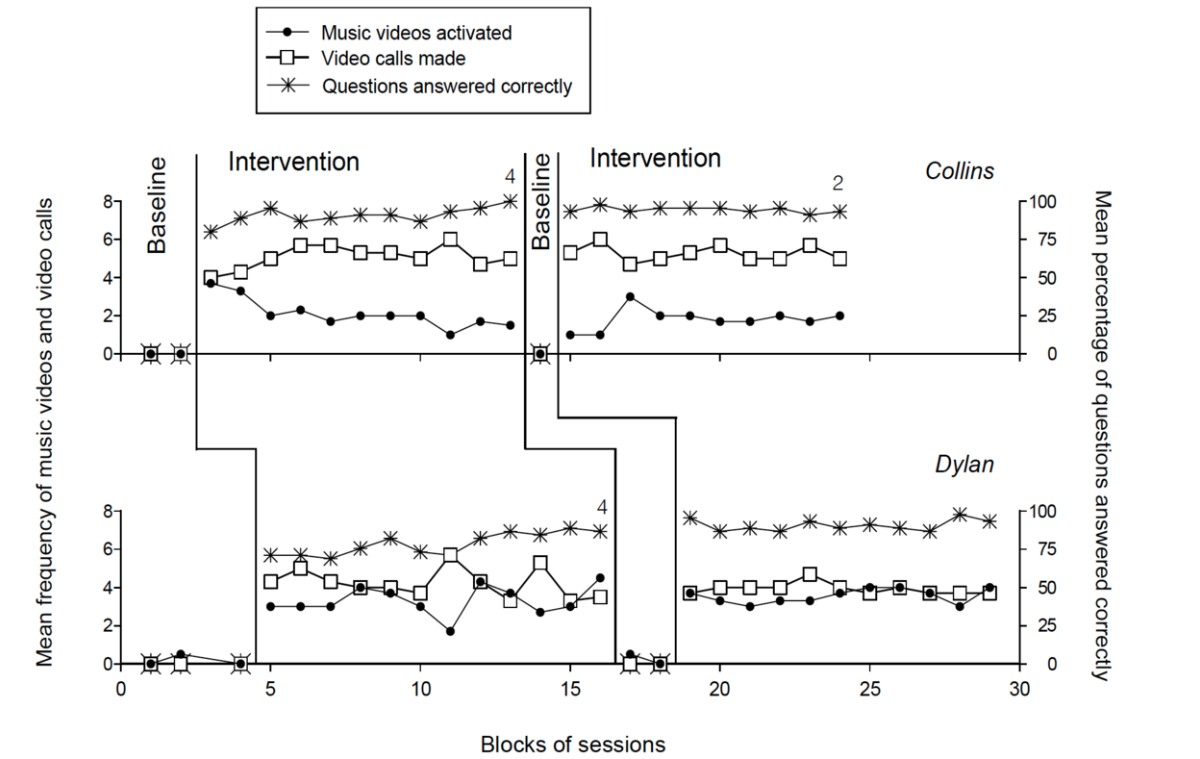
The 2 panels summarize the baseline and intervention data for Collins and Dylan. The data are plotted as in Figure 1.

The 2 panels of Figure 2 summarize the baseline and intervention data for the participants involved in the second program who used Technology System II (ie, Collins and Dylan). The data are plotted as in Figure 1, but the black circles and empty squares represent music videos activated and telephone calls made, respectively.

The first baseline phase showed that Collins (who received 4 sessions within 1 week) did not manage any form of response. Dylan (who received 6 sessions spread over 2 weeks) activated 1 music video. The number of baseline sessions used for Collins was limited (in this baseline phase as well as in the second; see below) because of her clearly insufficient skills to use the tablet and her related frustration. During the 34 (Collins) and 37 (Dylan) sessions of the first intervention phase, the mean frequency of music videos activated per session was 2.1 and 3.3, respectively. Their mean frequency of video calls made per session was 5.1 and 4.2, respectively. This frequency also includes video calls without a response from the partner (ie, calls in which the tablet played a prerecorded message of the partner called). Their mean percentage of correct story-related responses was 91 and 79, respectively. Their mean session duration was about 26 and 28 minutes, respectively. The data of the second baseline phase (including 2 and 4 sessions, respectively) and the second intervention phase (including 29 and 33 sessions, respectively) were similar to those obtained during the first baseline and intervention phases.

Comparisons made between intervention and baseline data through the PND method on each of the measures provided indices of 1.0 with an exception. This concerned the songs activated measure, on which Collins had an index of 0.95 (ie, an index that still expresses a strong intervention effect [45]).

## Discussion

### Principal Findings

The findings suggest that the new technology-aided programs were helpful for enabling the participants to independently activate preferred songs or music videos, send and receive voice messages or make video calls, and listen to brief stories and answer related questions. These findings, which need to be interpreted with caution given the nature of the study and the small number of participants, seem to extend the evidence of previous work focused on helping people with motor, sensory, and intellectual (cognitive) disabilities manage multiple forms of functional occupation [11,34,37,46]. Indeed, they seem to indicate that (1) various technology solutions might be profitably arranged to address different participants’ needs; (2) programs might be set up to include a form of cognitive exercise (ie, listening to brief stories and responding to questions about the stories) for participants who would have serious difficulties engaging in practical occupational tasks; and (3) voice messages might be used to allow participants, who have basic speech skills but are not keen on telephone calls, to have a personalized (emotionally direct) form of communication with their preferred partners. In light of the above, a number of considerations would seem pertinent.

First, technology systems that are simple, based on commercially available devices, and able to support intervention programs for people with different needs might be viewed as fairly practical (suitable) for rehabilitation contexts [47,48]. In this study, 2 such technology systems were evaluated for allowing people with different characteristics to reach comparable goals. The smartphone combined with the Bluetooth Blue2 switch appeared helpful for participants who were blind, but had a level of hand control that allowed them to use the pressure buttons of the Bluetooth Blue2 switch. The tablet interfaced with the buddy buttons seemed adequate for participants who possessed functional vision but had no or poor hand control, and therefore needed the buddy buttons’ position to be adapted to their plausible response mode (ie, at the wheelchair’s headrest or at different points of the desk).

Second, communication with distant partners may take different forms depending on the participants’ skills and preferences and the technology solutions available in the program [35,46]. In this study, voice messages were used with the first pair of participants whose verbal skills were not sufficient to successfully activate the smartphone’s Google Assistant, but were adequate to record and send voice messages comprehensible to the preferred communication partners. It was also thought that voice messages could represent a fairly personal and emotionally relevant form of communication for the sender and the receiver [49,50]. Video calls were used with the second pair of participants who had functional vision and were keen on this type of communication interaction.

Third, listening to smartphone or tablet presentations of brief stories and answering story-related questions represents a type of engagement that may be rather infrequent for participants with multiple disabilities [19-22]. Yet, such an engagement might be a meaningful alternative to other forms of occupation, such as practical daily activities, which are impossible or difficult to manage for participants with motor or visual and motor impairments. The same engagement might also be helpful to stimulate the participants’ attention and memory and thus might have a positive impact on their cognitive functioning [19,20].

Fourth, while these preliminary findings seem to be promising as to the impact of the programs, some clarification may be needed with regard to the programs’ applicability and costs. Regarding applicability, it may be noted that both programs rely on the use of a small number of commercially available devices that are easily portable and probably acceptable within daily contexts [47,51-53]. The cost of the technology systems used for the programs is about or slightly more than US $500. This includes about US $200 or $250 for the smartphone and about US $250 for the Bluetooth Blue2 switch (Technology System I), and about US $250 or $300 for the tablet, $150 for the Bluetooth Encore plus interface, and $120 for the 2 buddy sensors (Technology System II). The cost of the MacroDroid app is practically insignificant.

### Limitations

Three main limitations of the study can be underlined. The first limitation concerns the fact that only 2 participants were involved in each program. Based on this, the study as a whole can be viewed as preliminary, as a proof of concept, rather than as a definite demonstration of the ultimate value of the programs investigated [40,41,52]. Replication studies with new participants would be crucial to ascertain the strength and generality of the data obtained with the 2 programs and the feasibility of improving the programs [54-56]. The use of a multiple probe design without a withdrawal (second baseline) phase for the first pair of participants might technically be viewed as a methodological weakness [57]. In practice, however, the second baseline could hardly be considered a methodologically indispensable condition with those participants given their well-known and consolidated speech difficulties [37,58,59]. Indeed, one would not have expected the participants to improve their speech skills and become efficient in activating the smartphone’s Google Assistant through their utterances. This point (ie, lack of speech improvement) was documented with the second pair of participants for whom a second baseline phase was carried out after an intervention period.

A second limitation concerns the absence of any specific assessment of the participants’ satisfaction with (enjoyment of) their program. While anecdotal reports suggest that the participants wanted to be involved in the program sessions and were happy to access their preferred music and contact their preferred communication partners, a direct evaluation of their satisfaction with the program would be highly desirable. Such evaluation could involve 2 main steps. One step could consist of asking them to make choices between program sessions and some other form of daily engagement considered to be pleasing for them [60]. Another step could be to compare their mood expressions (eg, indices of happiness) during the program sessions and during other daily engagement situations [34,61,62].

A third limitation concerns the fact that no social validation of the programs was carried out. While the staff initially interviewed had expressed support for the programs and the technology involved (see the “Participants” section), a more specific and wider validation process should be pursued. Such validation could be carried out by asking groups of staff personnel familiar with this population to watch short videos of participants using the programs and then rate the programs’ friendliness, relevance, and applicability [63,64].

### Conclusions

The findings, which need to be interpreted with caution given the nature of the study and the small number of participants, seem to suggest that the new programs may be suitable to help people with motor or visual-motor and intellectual disabilities independently access functional forms of occupation and communication. Notwithstanding the encouraging findings, general statements about the programs and their overall implications for daily contexts must await the outcome of new research directed at replicating and extending this study and overcoming its limitations.

## Abbreviations

PND: percentage of non-overlapping data
SIM: subscriber identity module

## Data Availability

Data are available on the Open Science Framework [65].
